# EUS features and clinical outcomes of gastric calcifying fibrous tumor: a retrospective analysis of 20 cases

**DOI:** 10.3389/fonc.2026.1835347

**Published:** 2026-08-26

**Authors:** Bing Bai, Qiuyu Liu, Bingxi Zhou, Baosong Liang, Shengli Kuang

**Affiliations:** 1 Oncology Center, Henan Provincial People’s Hospital (People’s Hospital of Zhengzhou University), Zhengzhou, China; 2 Department of Pathology, Henan Provincial People’s Hospital (People’s Hospital of Zhengzhou University), Zhengzhou, China; 3 Department of Gastroenterology, Henan Provincial People’s Hospital (People’s Hospital of Zhengzhou University), Zhengzhou, China

**Keywords:** endoscopic resection, endoscopic ultrasonography, gastric calcifying fibrous tumor, muscularis propria, subepitheial lesion

## Abstract

**Objective:**

To analyze the endoscopic ultrasonography (EUS) features and clinical characteristics of gastric calcifying fibrous tumor (GCFT) to improve the endoscopic recognition of this entity.

**Methods:**

A retrospective analysis was conducted on 20 consecutive patients with pathologically confirmed GCFT at Henan Provincial People’s Hospital from January 2018 to May 2025. EUS findings and clinical data were reviewed to summarize the endoscopic and clinical features of this rare lesion.

**Results:**

Of the 20 patients, 12 (60.0%) were female, and 8 (40.0%) were male, with a mean age of 46.65 years (range: 29–62 years). Tumor size ranged from 0.5 to 2.8 cm, with a mean diameter of 1.18 ± 0.55 cm. Most tumors were located in the gastric body (n = 16), followed by the fundus (n = 4). On computed tomography, most lesions presented as solid hyperdense nodules within the gastric wall. Endoscopically, all lesions appeared as submucosal tumors with smooth overlying mucosa. Preoperative EUS showed hypoechoic lesions in 19 cases and iso- to hypoechoic lesions in one. All originated from the muscularis propria. Hyperechoic foci, suggestive of calcifications, were observed in 13 lesions (central calcification in 1, peripheral in 12), accompanied by posterior acoustic shadowing. The overlying mucosa and submucosa appeared thickened compared to the surrounding normal layers. All patients underwent endoscopic resection. Histopathologically, tumors consisted of abundant hyalinized collagenous tissue with occasional calcifications and psammoma bodies. The Ki-67 index ranged from <1% to 5%, and immunohistochemical staining was negative for S-100, CD34, CD117, and Desmin. During a follow-up period of 5 to 71 months, no recurrence was observed.

**Conclusion:**

GCFT typically presents as a hypoechoic mass originating from the muscularis propria on EUS, frequently accompanied by calcification and deep echo attenuation. These features, when present, may suggest the diagnosis. Endoscopic resection is safe and effective for selected lesions.

## 1 Introduction

Gastric calcifying fibrous tumor (GCFT) is a rare mesenchymal tumor of the stomach characterized by low incidence, lack of typical clinical manifestations, and difficulty in preoperative diagnosis (1, 2). With advances in imaging and endoscopic diagnostic techniques, clinical attention to GCFT has been increasing. This study retrospectively reviewed data from 20 patients with GCFT, analyzed endoscopic ultrasonography (EUS) findings and clinical characteristics, and summarized commonly observed EUS features that may aid in its recognition. A better understanding of these features may contribute to the differential diagnosis from other subepithelial lesions and is crucial for selecting appropriate treatment strategies.

## 2 Materials and methods

### 2.1 Study population

Data were prospectively collected from 20 consecutive patients with pathologically confirmed gastric calcifying fibrous tumor (GCFT) who underwent endoscopic resection (ESD or EFR) at the Endoscopy Center of Henan Provincial People’s Hospital between January 2018 and May 2025. Inclusion criteria were: (1) pathologically confirmed GCFT based on histology and immunohistochemistry; (2) preoperative EUS evaluation performed; (3) complete clinical, endoscopic, and follow-up data available. Exclusion criteria included: (1) lesions managed by surgery or observation only; (2) incomplete medical records. All pathological specimens were independently reviewed by two senior gastrointestinal pathologists to confirm the diagnosis.

### 2.2 EUS examination and image analysis

EUS was performed using a radial scanning echoendoscope (EG-3670URK, Pentax, Japan) or a 12/20-MHz miniprobe. All procedures were performed by experienced endoscopists with over 5 years of experience in EUS. Stored EUS images were retrospectively reviewed from the hospital PACS by the same operators. The following EUS features were assessed and defined:

Origin layer: The layer of the gastric wall from which the lesion arose.

Echogenicity: Compared to the adjacent muscularis propria; categorized as hypoechoic, isoechoic, or hyperechoic.

Calcification: Presence of hyperechoic foci, with or without posterior acoustic shadowing.

Deep echo attenuation: Significant reduction in echo amplitude distal to the lesion.

Mucosal-submucosal thickening: Visibly increased thickness of the mucosal and submucosal layers overlying the lesion compared to adjacent normal tissue.

### 2.3 Statistical analysis

Descriptive statistical analysis was employed. Continuous variables are presented as mean ± SD or median (range), and categorical variables as frequencies and percentages.

## 3 Results

### 3.1 Clinical characteristics

**Figure 1:**
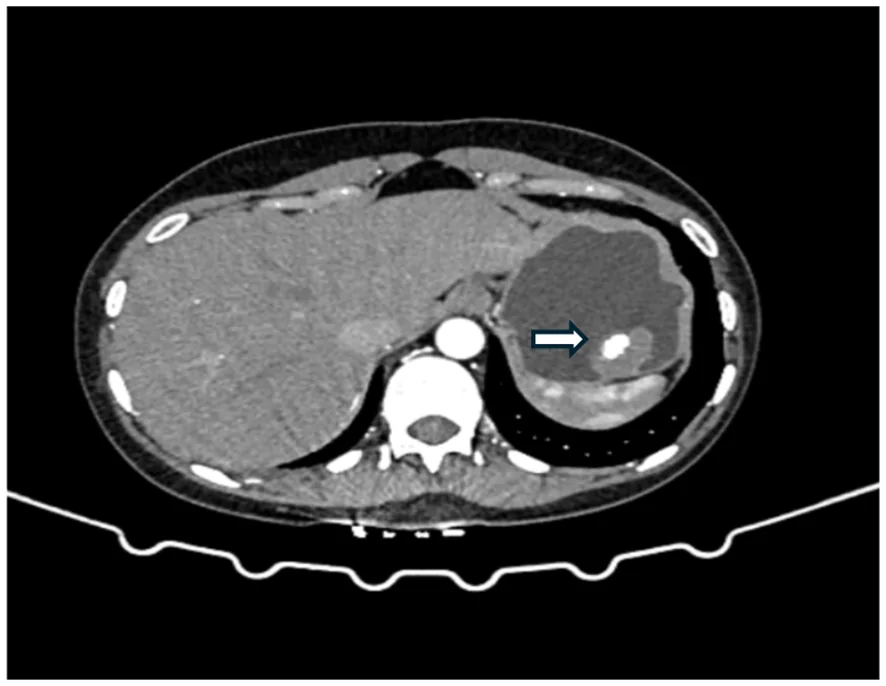
CT shows a high-density nodule with internal calcifications(**arrow**).

**Figure 2:**
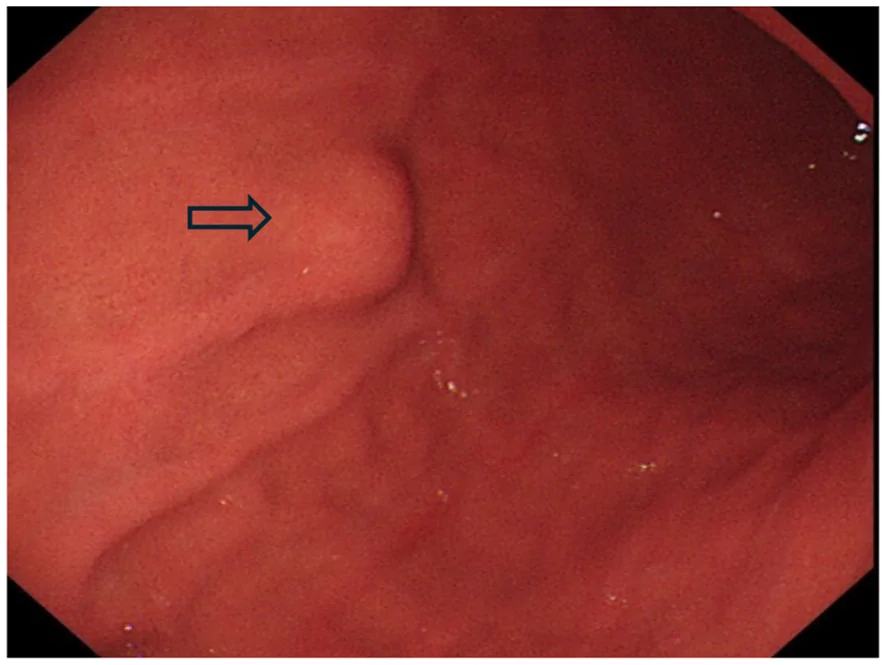
Gastroscopy reveals a submucosal mass with a smooth mucosal surface (arrow).

This study describes the clinical features of 20 cases of gastric CFT (**Tables 1**, **2**). There were 12 female patients (60.0%) and 8 male patients (40.0%); ages ranged from 29 to 62 years, with a mean age of 46.65 ± 10.00 years. Tumor longest diameter ranged from 0.5 to 2.8 cm, with a mean longest diameter of 1.2 ± 0.54 cm. The majority of tumors were located in the gastric body (16 cases, 80%), followed by the fundus (4 cases, 20%). In terms of distribution, 8 cases were on the greater curvature side, with involvement also noted on the lesser curvature, and anterior wall. Imaging CT findings predominantly showed nodular hyperdense lesions; some cases demonstrated calcification (**Figure 1**). Endoscopic examination revealed subepithelial lesions with smooth overlying mucosa (**Figure 2**). Further endoscopic resection was performed [endoscopic full-thickness resection (EFR) in 6 cases, endoscopic submucosal dissection (ESD) in 14cases].

**Table 1:** Clinical, endoscopic, and EUS characteristics of 20 patients with gastric calcifying fibrous tumor.

ID	Gender	Age (y)	Location	Orientation	Diameter (cm)	Endoscopic findings	Origin layer	Echogenicity	Central calc.	Peripheral calc.	Far-field pattern	Mucosal-submucosal thickening	CT findings	Symptoms	Procedure	F/U (mo)
1	Female	40	Body	Greater Curvature	1.3 × 0.8	Non-pedunculated, broad-based elevation	Muscularis Propria	Hypoechoic	No	Yes	Attenuation	Positive	Submucosal lesion in the greater curvature of the gastric body	Intermittent upper abdominal distension and acid regurgitation	ESD	71
2	Female	46	Gastric Body	Greater Curvature	1.0 × 1.0	Non-pedunculated, broad-based elevation	Muscularis Propria	Hypoechoic	No	Yes	Attenuation	Positive	Mass with calcification in the greater curvature of the gastric body	Intermittent abdominal distension and pain	ESD	69
3	Male	40	Body	Lesser Curvature	1.2 × 1.2	Non-pedunculated, broad-based elevation	Muscularis Propria	Hypoechoic	No	Yes	Attenuation	Positive	Nodular high-density lesion in the gastric wall	Physical examination	ESD	64
4	Female	49	Fundus	Greater Curvature	1.0 × 0.8	Non-pedunculated, broad-based elevation	Muscularis Propria	Hypoechoic	No	Yes	Attenuation	Positive	Nodule with calcification in the gastric wall	Intermittent abdominal distension	EFR	62
5	Male	39	Body	Lesser Curvature	0.9 × 0.8	Spherical elevation	Muscularis Propria	Hypoechoic	No	Yes	Attenuation	Positive	Nodular high-density lesion in the gastric wall	Upper abdominal distension	ESD	58
6	Female	38	Body	Anterior Wall	1.0 × 0.8	Non-pedunculated, broad-based elevation	Muscularis Propria	Hypoechoic	No	Yes	Attenuation	Positive	Solid nodule in the anterior wall of the gastric body	Pain under the xiphoid process	ESD	56
7	Male	29	Body	Posterior Wall	0.7 × 0.4	Non-pedunculated, broad-based elevation	Muscularis Propria	Hypoechoic	No	No	No attenuation	Positive	Not detected	Physical examination	ESD	51
8	Female	42	Body	Anterior Wall	2.0 × 1.0	Non-pedunculated, broad-based elevation	Muscularis Propria	Hypoechoic	No	Yes	Attenuation	Positive	Nodule in the anterior wall of the gastric body	Physical examination	EFR	43
9	Male	47	Fundus	Greater Curvature	1.2 × 0.7	Non-pedunculated, broad-based elevation	Muscularis Propria	Hypoechoic	No	Yes	Attenuation	Positive	Nodular high-density lesion in the fundus with local wall thickening	Physical examination	ESD	42
10	Female	61	Body	Greater Curvature	2.0 × 1.5	Non-pedunculated, broad-based elevation	Muscularis Propria	Hypoechoic	No	Yes	Attenuation	Positive	Solid nodule with calcification in the greater curvature of the gastric body	Abdominal pain	ESD	42
11	Female	51	Fundus	Greater Curvature	1.1 × 0.7	Non-pedunculated, broad-based elevation	Muscularis Propria	Hypoechoic	No	No	Attenuation	Positive	Nodule with calcification in the gastric wall	Intermittent abdominal distension, pain, and acid regurgitation	ESD	39
12	Male	62	Body	Greater Curvature	0.6 × 0.6	Non-pedunculated, broad-based elevation	Muscularis Propria	Hypoechoic	No	No	Attenuation	Positive	No abnormalities detected	Abdominal distension	ESD	38
13	Female	44	Body	Greater Curvature	1.2 × 1.1	Non-pedunculated, broad-based elevation	Muscularis Propria	Hypoechoic	No	No	Attenuation	Positive	Solid nodule in the greater curvature of the gastric body	Abdominal distension	EFR	37
14	Female	49	Body	Anterior Wall	1.5 × 1.5	Non-pedunculated, broad-based elevation	Muscularis Propria	Iso- to hypoechoic	No	Yes	Attenuation	Positive	Nodular high-density lesion in the greater curvature	Physical examination	EFR	36
15	Female	34	Fundus	Greater Curvature	2.8 × 2.1	Non-pedunculated, broad-based elevation	Muscularis Propria	Hypoechoic	No	Yes	Attenuation	Positive	Mass in the fundus of the stomach	Physical examination	EFR	35
16	Female	55	Body	Greater Curvature	0.5 × 0.4	Non-pedunculated, broad-based elevation	Muscularis Propria	Hypoechoic	No	No	No attenuation	Positive	Nodule with calcification in the gastric wall	Physical examination	ESD	32
17	Male	58	Body	Anterior Wall	1.2 × 0.8	Non-pedunculated, broad-based elevation	Muscularis Propria	Hypoechoic	Yes	No	Attenuation	Positive	Nodular abnormal enhancement in the greater curvature of the gastric body	Pain under the xiphoid process	ESD	28
18	Male	32	Body	Greater Curvature	1.0 × 0.8	Minimal elevation	Muscularis Propria	Hypoechoic	No	No	Attenuation	Positive	Solid nodule in the greater curvature of the gastric body	Abdominal pain, distension, and acid regurgitation	ESD	10
19	Female	56	Body	Greater Curvature	0.6 × 0.3	Non-pedunculated, broad-based elevation	Muscularis Propria	Hypoechoic	No	No	Attenuation	Positive	No abnormalities detected	Physical examination	ESD	8
20	Male	61	Body	Anterior Wall	0.8 × 0.6	Non-pedunculated, broad-based elevation	Muscularis Propria	Hypoechoic	No	Yes	Attenuation	Positive	Solid nodule in the anterior wall of the gastric body	Abdominal distension and pain	EFR	5

GCFT, gastric calcifying fibrous tumor; EUS, endoscopic ultrasound; ESD, endoscopic submucosal dissection; EFR, endoscopic full-thickness resection; F/U, follow-up.

Calcification: presence of hyperechoic foci with or without posterior acoustic shadowing. Deep echo attenuation: significant reduction in echo amplitude distal to the lesion. Mucosal-submucosal thickening: visibly increased thickness of the mucosal and submucosal layers overlying the lesion compared to adjacent normal tissue.

All 20 specimens were independently reviewed by two senior gastrointestinal pathologists.

**Table 2:** Summary of clinical, EUS, pathological, treatment, and follow-up findings.

Characteristic	Value (n=20)
Total patients, n	20
Female/Male, n (%)	12 (60.0)/8 (40.0)
Mean age ± SD (years)	46.65 ± 10.00
Age range (years)	29–62
Location, n (%)	
Body	16 (80.0)
Fundus	4 (20.0)
Mean diameter ± SD (cm)	1.18 ± 0.54
Diameter range (cm)	0.5–2.8
Origin: Muscularis Propria, n (%)	20 (100)
Echogenicity, n (%)	
Hypoechoic	19 (95.0)
Iso- to hypoechoic	1 (5.0)
Peripheral calcification, n (%)	12 (60.0)
Central calcification, n (%)	1 (5.0)
No calcification, n (%)	7 (35.0)
Deep echo attenuation, n (%)	18 (90.0)
Mucosal-submucosal thickening, n (%)	20 (100)
En bloc resection rate, n (%)	20 (100)
R0 resection rate, n (%)	18 (90.0)
ESD, n (%)	14 (70.0)
EFR, n (%)	6 (30.0)
Procedure-related bleeding, n (%)	2 (10.0)
Perforation, n (%)	0 (0)
Median follow-up (months)	18 (range 5–71)
Recurrence, n (%)	0 (0)

### 3.2 Endoscopic ultrasound characteristics

**Figures 3:**
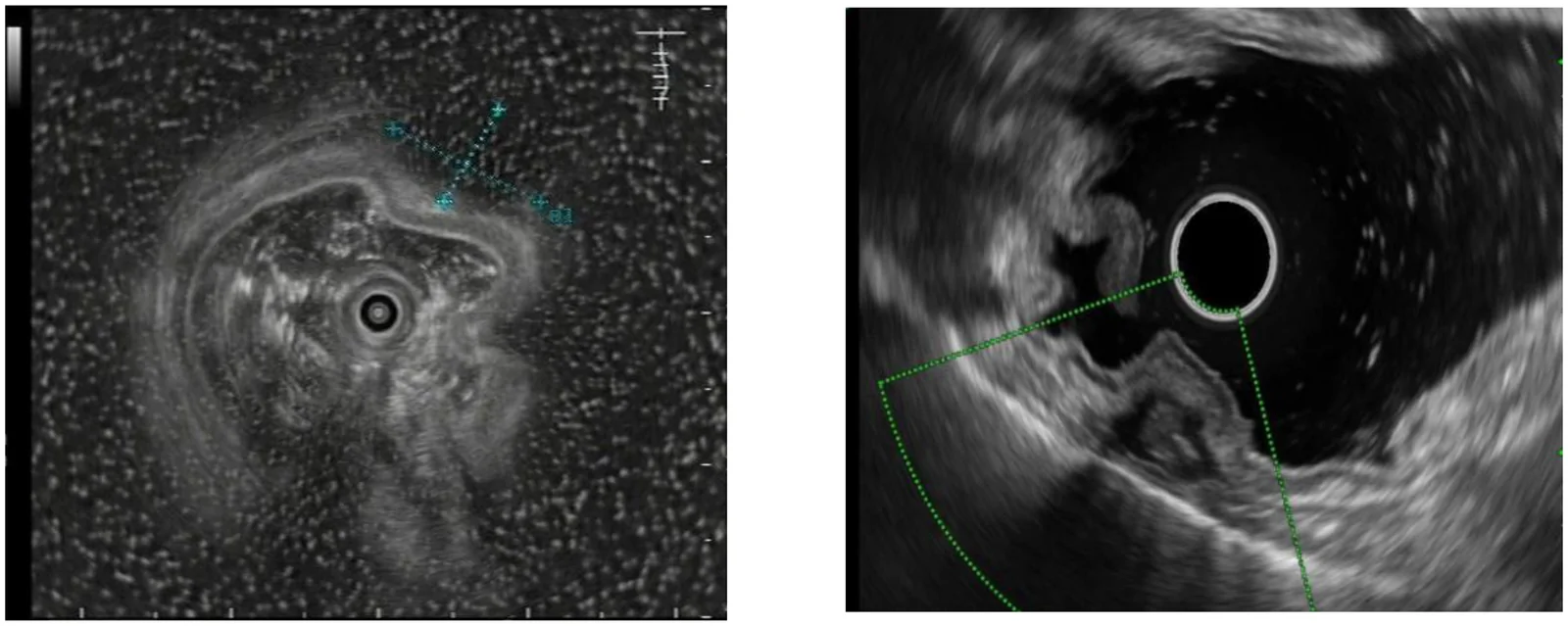
The mucosal-submucosal layer exhibits increased thickness compared to the surrounding mucosal and submucosal layers. The tumor originates from the muscularis propria, with deep echo attenuation.

1. Size and Layers: Tumor sizes varied significantly, with the longest diameters ranging from 0.5 cm to 2.8 cm. All tumors originated from the muscularis propria.
2. Acoustic characteristics: 19 cases (95.0%) exhibited hypoechoic patterns, while 1 case (5.0%) showed meso-hypoechoic characteristics. Deep echo attenuation was observed in 18 cases (90%). Central calcification was present in 1 case, and peripheral calcification in 12 cases (total 13 cases, 65.0%). Detailed analysis of sonographic features revealed increased mucosal-submucosal layer thickness at lesion surfaces compared to surrounding mucosal and submucosal layers. (**Figure 3**).

### 3.3 Treatment outcomes and follow-up

All 20 patients underwent successful endoscopic resection. The en bloc resection rate was 100% (20/20), and the R0 resection rate was 90.0% (18/20). Procedure-related bleeding occurred in 2 cases (10.0%), both managed endoscopically. No perforation or surgical conversion occurred. The median length of hospital stay was 4 days (range 3–7 days). During a median follow-up of 18 months (range 5–71 months), no recurrence was observed.

### 3.4 Pathological features

**Figure 4:**
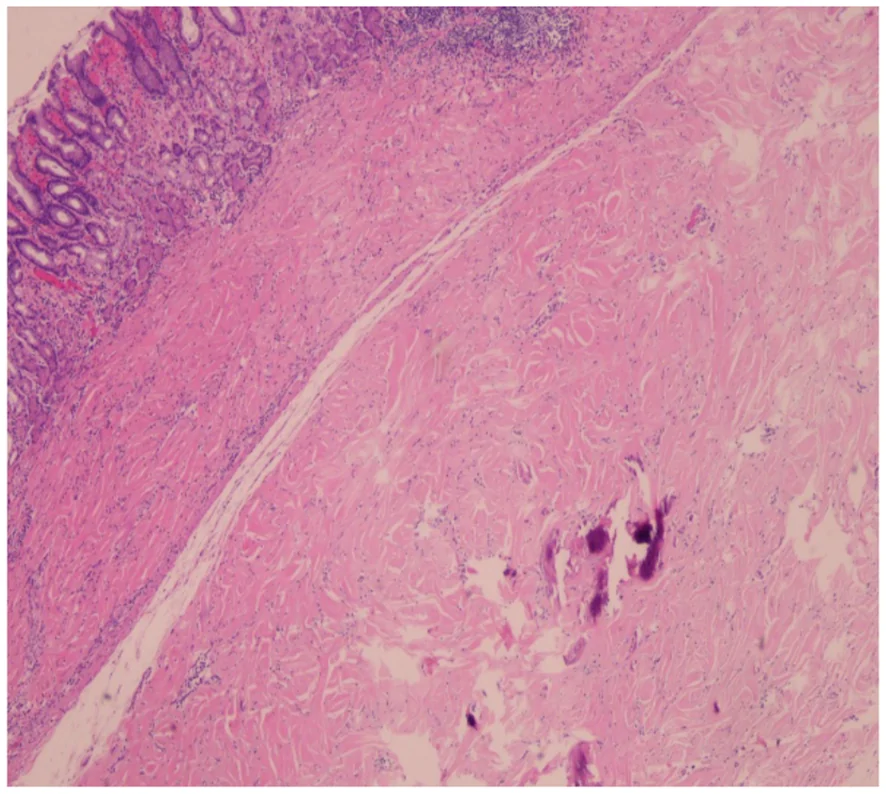
The mucosal layer and submucosal layer on the tumor surface remain intact, with well-defined tumor margins.

**Figure 5:**
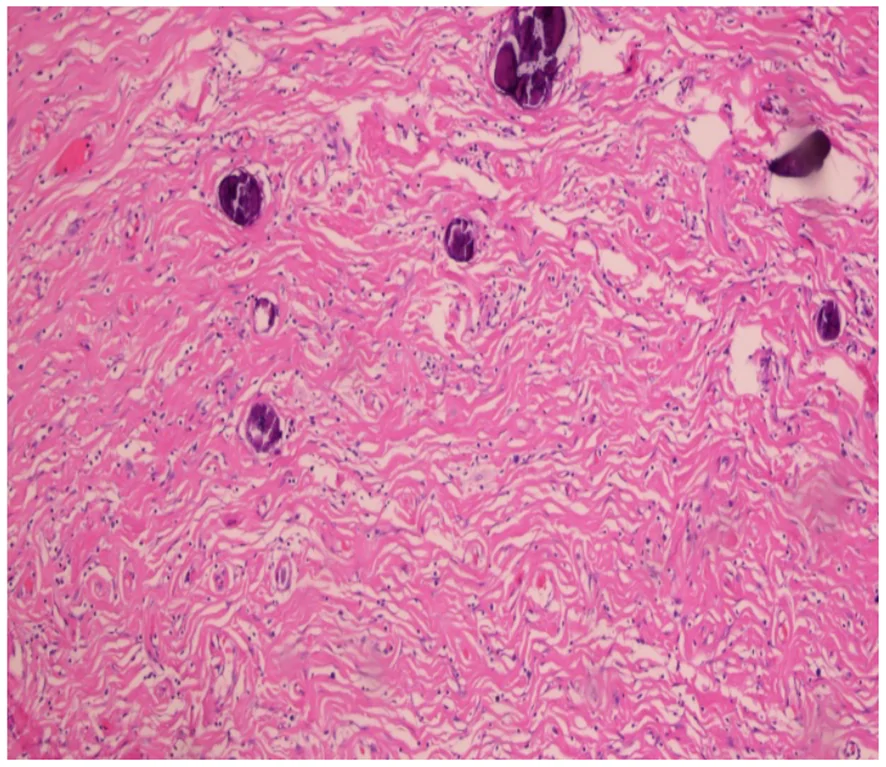
A small number of spindle-shaped fibroblasts are interspersed within a background of collagenized fibrous connective tissue, with calcifications or granulation bodies visible.

The 20 tumors measured approximately 0.5–2.8 cm in longest diameter, presenting as oval or irregular nodular masses without distinct capsules. Cross-sections appeared grayish-white, firm in consistency, with well-defined margins. Microscopically, a background of extensively collagenized fibrous connective tissue contained scattered spindle-shaped fibroblasts, along with calcifications or psammoma bodies. The stroma showed variable infiltration by lymphocytes and plasma cells (**Figures 4**, **5**). In this study, 11 cases showed lymphocyte aggregation and lymphoid follicle formation within the stroma. Immunohistochemistry: Ki-67 index ranged from <1% to 5%. Spindle cells were positive for vimentin, while SMA, CD117, CD34, DOG1, S-100, desmin, and ALK were all negative.

## 4 Discussion

CFT is an extremely rare benign fibrous tumor primarily affecting children and adolescents. It commonly occurs in the subcutaneous or deep soft tissues of the extremities, trunk, groin, and head and neck regions (2, 3). As a rare mesenchymal-derived benign tumor of the stomach, GCFT has been predominantly reported in case series, lacking large-scale cohort analyses (4, 5). This study systematically reviews the clinical, imaging, and pathological characteristics of 20 cases of GCFT, providing more targeted practical references for the precise diagnosis and treatment of this rare benign tumor.

Among the 20 cases of GCFT in this study, females slightly outnumbered males, accounting for 60.0%. This finding aligns with gender distribution reported in some literature (6).

For subepithelial lesions of the stomach, conventional imaging studies such as CT and MRI can detect space-occupying lesions, providing preliminary indications of lesion location and size (1, 7). As the gold standard for evaluating subepithelial lesions, EUS holds irreplaceable core significance in localization, characterization, and treatment planning (8). All 20 GCFT cases in this study underwent EUS examination.

In this study, EUS examination confirmed that all 20 cases of GCFT originated from the muscularis propria. This finding revealed the core anatomical characteristics of GCFT and provides critical evidence for selecting subsequent endoscopic treatment strategies (ESD or EFR). EUS clearly displays key characteristic features of GCFT, including internal echo patterns, margins, and calcifications. The most commonly observed EUS features were: (1) origin in the muscularis propria; (2) predominantly hypoechoic pattern (95.0%); (3) calcification (65.0%); and (4) deep echo attenuation (90.0%). These features, when present together, may raise suspicion for GCFT. However, we acknowledge that these findings require validation in comparative studies including Gastrointestinal stromal tumors (GISTs), gastric leiomyomas, and other subepithelial lesions.

Importantly, this study also identified a previously underreported subtle feature: increased mucosal-submucosal thickness at the surface of GCFT lesions compared to surrounding areas. This finding adds a new dimension to the differential diagnosis of subepithelial lesions, further highlighting the diagnostic advantages of EUS in capturing subtle anatomical details.

The histopathological features of GCFT reveal that the mucosal layer and submucosal layer of the tumor surface remain intact without compression, with well-defined tumor margins. These findings closely align with its characteristics observed under EUS, thereby enhancing preoperative diagnostic accuracy. Simultaneously, these features aid in differentiation from other subepithelial lesions. For example, GISTs also frequently originate in the muscularis propria, but they are typically immunoreactive for CD117, which is a key diagnostic marker distinguishing them from GCFT (which is typically CD117-negative). Smooth muscle tumors typically exhibit positive staining for SMA and Desmin.

The study has certain limitations: as a single-center retrospective analysis, the sample size—though larger than previous case reports—remains relatively small and may introduce selection bias. Further validation requires multicenter, prospective studies. Additionally, the absence of a control group of other subepithelial lesions (e.g., GISTs, leiomyomas) means that the specificity of the reported EUS features for GCFT cannot be determined from this study alone. Currently, ESD and EFR are safe and effective treatments for GCFT and can serve as the preferred therapeutic options. Enhancing clinical awareness of this disease, particularly its characteristic EUS manifestations, combined with multimodal imaging and pathological examination, enables precise diagnosis and treatment, thereby improving patient prognosis.

## 5 Conclusion

GCFT is a rare benign gastric mesenchymal tumor that should be considered in the differential diagnosis of gastric subepithelial lesions. On EUS, it commonly presents as a hypoechoic mass originating from the muscularis propria, frequently accompanied by calcification and deep echo attenuation. Endoscopic resection (ESD or EFR) appears to be a safe and effective treatment option for selected lesions, with no recurrence observed during the available follow-up period. Further comparative studies are needed to validate the diagnostic utility of these EUS features.

## Data Availability

The original contributions presented in the study are included in the article/supplementary material, further inquiries can be directed to the corresponding author.
